# Supplementary material for: TandemTwister: scalable genotyping and advanced visualization of tandem repeats
Source: NAR Genom Bioinform. 2026 Aug 17;8(3):lqag096. doi: 10.1093/nargab/lqag096 (PMC13478753; doi:10.1093/nargab/lqag096)
Supplement: Supplementary file 1 [file lqag096_supplemental_file.pdf]

Supplementary Information for: [TandemTwister: Scalable genotyping and advanced visualization of tandem repeats]

Lion Ward Al Raei[1,2†], Maryam Ghareghani[1,2†], M-Hossein Moeinzadeh[1,3], and Martin Vingron[1,2#]

[†] *equal contribution*
[#] *corresponding author*
[1] *Department of Computational Molecular Biology, Max Planck Institute for Molecular Genetics, Berlin, Germany.*
[2] *Department of Mathematics and Computer Science, Freie Universität Berlin, Germany.*
[3] *Lucid Genomics, Berlin, Germany.*

#Contact: vingron@molgen.mpg.de

Gene	Disease	Motif	Pathogenic threshold	Region
DAB1	Spinocerebellar Ataxia Type 37	GAAAT	31.0	chr1:57367044-57367125
NOTCH2NLC	Neuronal intranuclear inclusion disease, Alzheimer disease and parkinsonism phenotype	GGC	66.0	chr1:149390803-149390842
HTT	Huntington disease	CAG	40.0	chr4:3074877-3074940
RFC1	Cerebellar ataxia, neuropathy, and vestibular areflexia syndrome	AAGGG, ACAGG, AGGGC, AAGGC, AGAGG	400.0	chr4:39348425-39348483
ATXN1	Spinocerebellar Ataxia Type 1	CTG	39.0	chr6:16327634-16327724
FXN	Friedreich ataxia	GAA	56.0	chr9:69037286-69037304
PABPN1	Oculopharyngeal muscular dystrophy	GCA, GCT, GCC, GCG	12.0	chr14:23321472-23321502
DMPK	Myotonic Dystrophy Type 1	CAG	50.0	chr19:45770204-45770266
FMR1	Fragile X syndrome (FXS)	CGG	201.0	chrX:147912037-147912111

Supplementary Table S1: Repeat expansion disorders. Disease-associated tandem repeat loci analyzed in this study, including the repeat motif, pathogenic expansion threshold, and genomic region for each gene. Thresholds are based on STRchive [Hiatt et al., 2025].

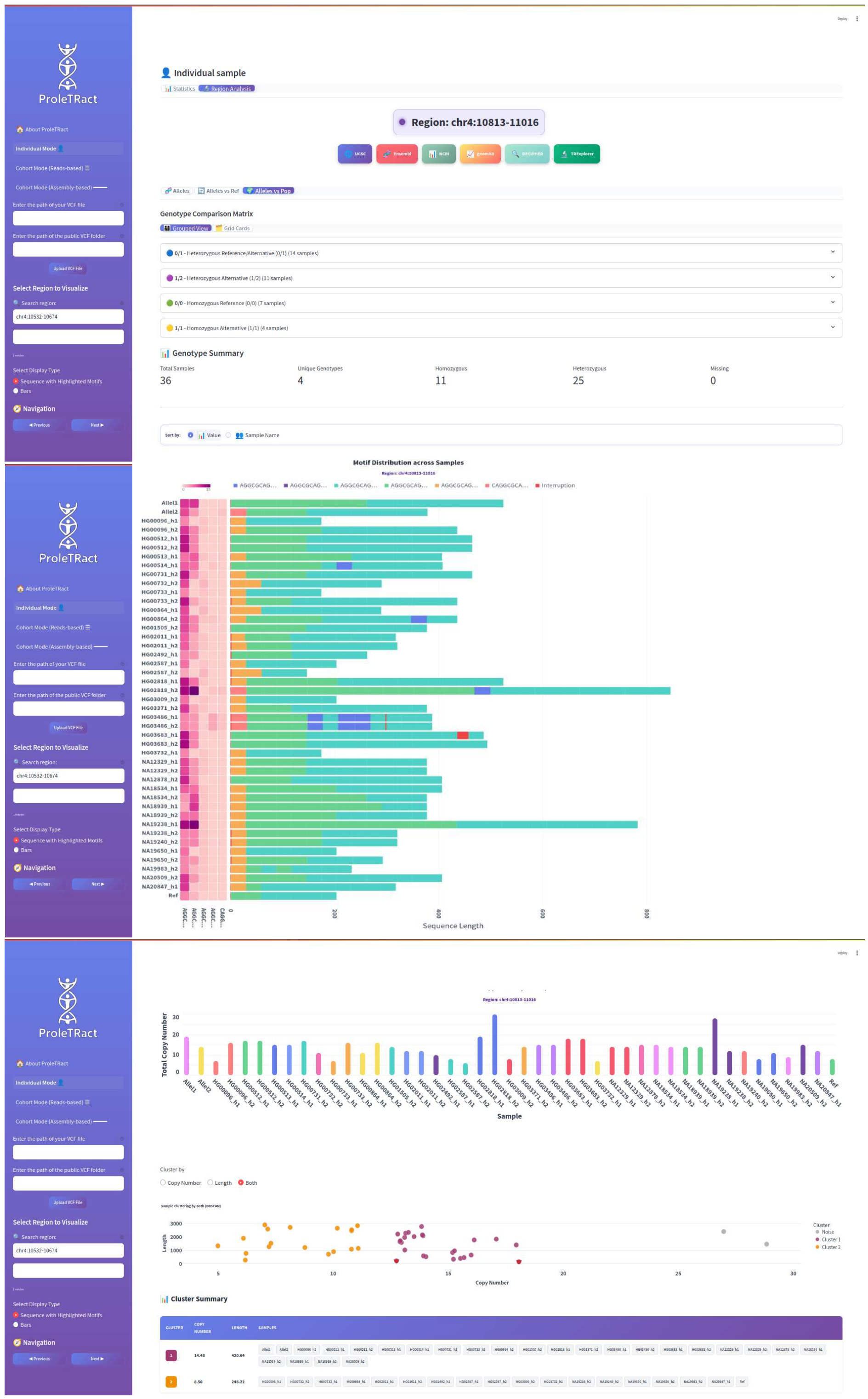

Supplementary Figure S1: Overview of ProleTRact's UI. The following features are displayed: Top panel: optional buttons for redirecting the user to differet views of the tandem repeat region in public datasets and genome browsers such as USCS, ensemble, etc. Information and groups of genotypes for popualtion samples, together with summary counts for uniques, homozygous and heterozygous samples. Middle panel: population-level visualisation, including heatmaps displaying different motif counts in the population samples, stacked bar plots of motif occurences in each haplotype sample. Bottom panel: population-level visualisation, including heatmaps bar plots of total motif counts per sample and clustering samples by their motif copy number and repeat length features. A clustering summary is also provided by listing the names of samples falling into different clusters and their corresponding copy number and length range.

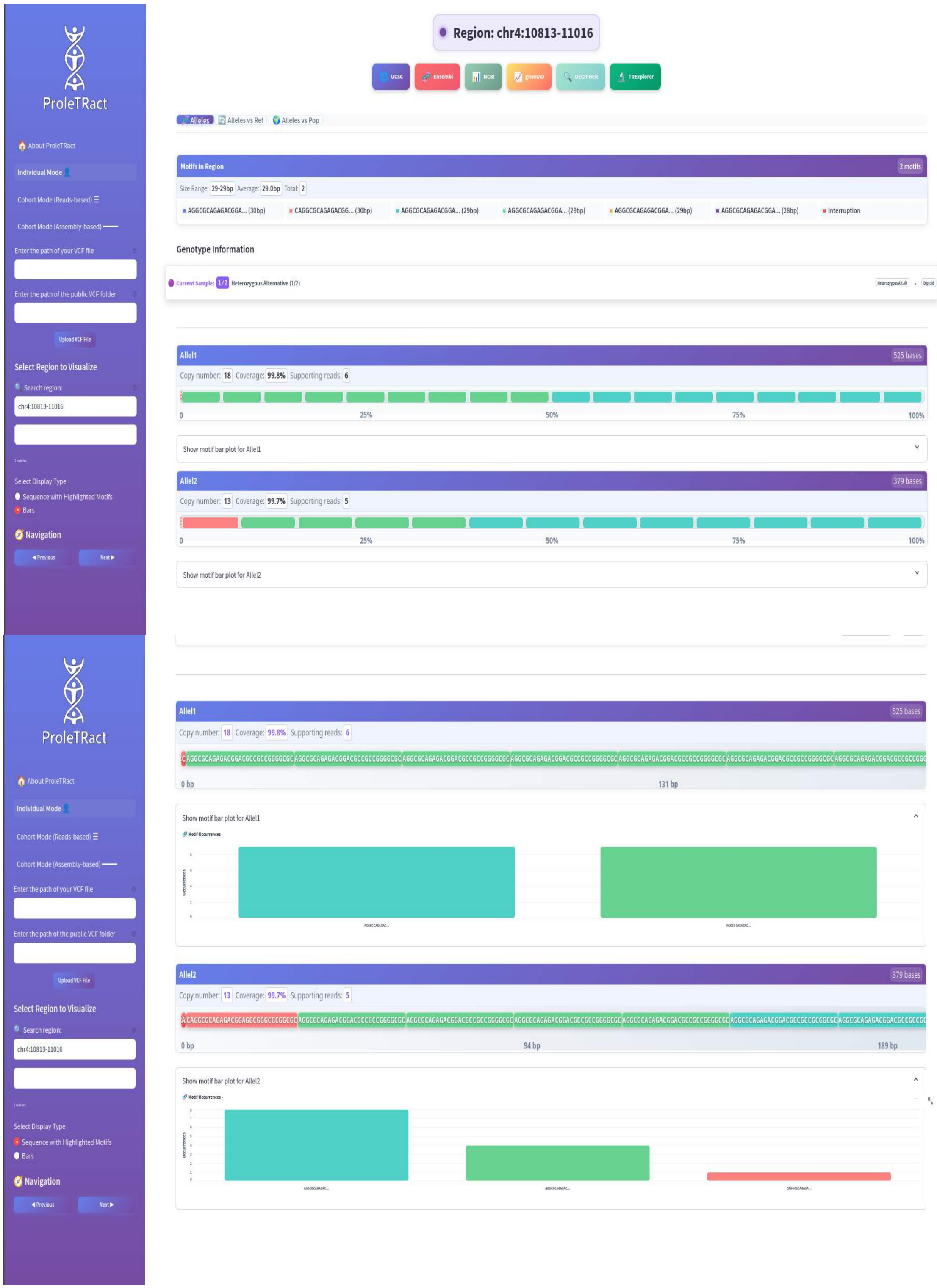

Supplementary Figure S2: Overview of ProleTRact's UI. The following features are displayed: Top panel: optional buttons for redirecting the user to differet views of the tandem repeat region in public datasets and genome browsers such as USCS, ensemble, etc. Visualisation of motif compositions in tandem repeat alleles by colored bars including the totl copy number and read coverage information. Bottom panel: Color-coded bar plots representing individual motif copy numbers of each allele.

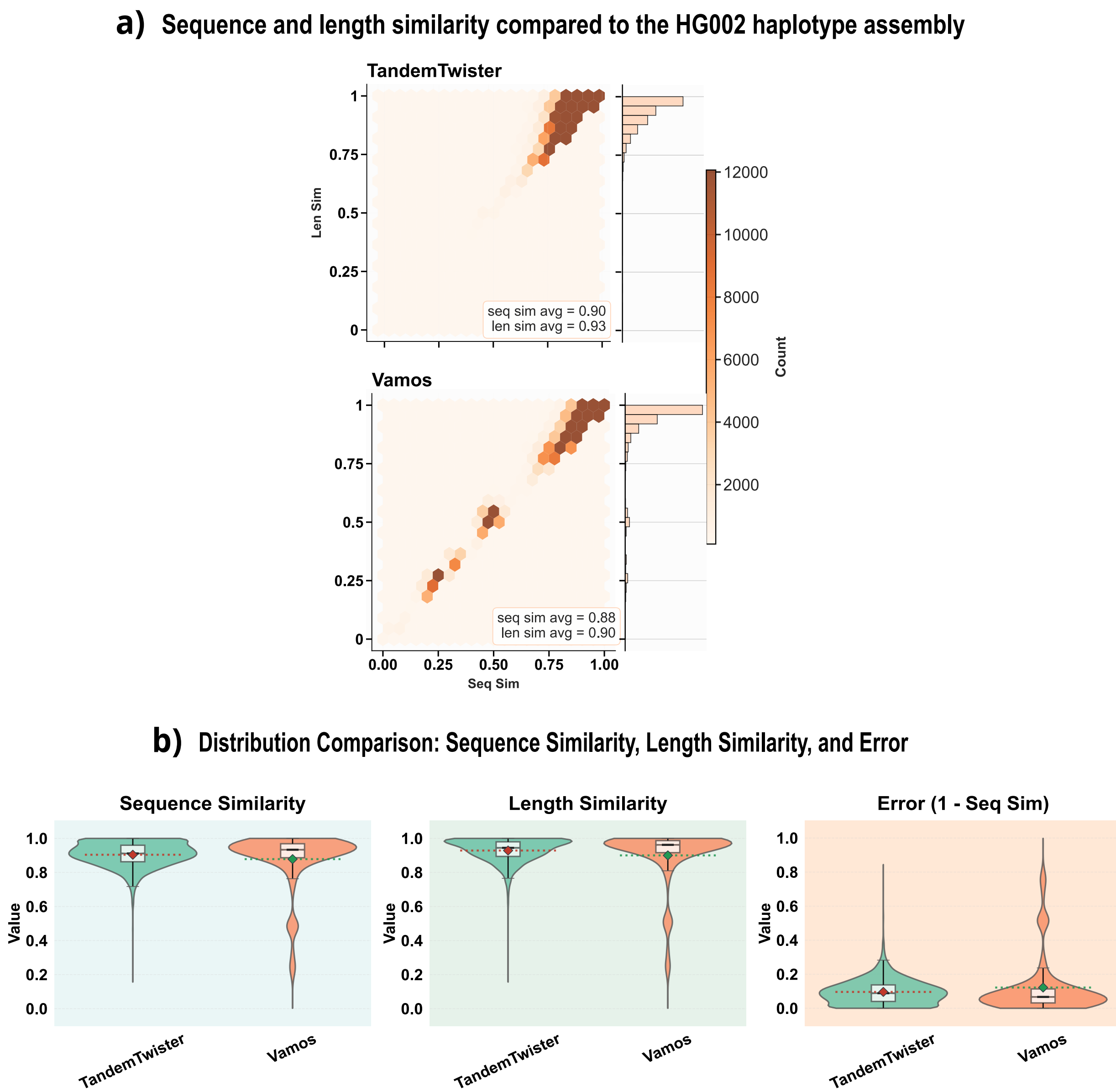

Supplementary Figure S3: Sequence accuracy on PacBio CLR sample HG002. a) Comparison of the concatenated TR allele sequences with the high-quality haplotype assemblies. Hexa plots representing density of existing regions with various sequence and length similarities in the two tools. b) Comparison of different tool's distributions of sequence similarity (left), length similarity (middle) and sequence error rate (right).

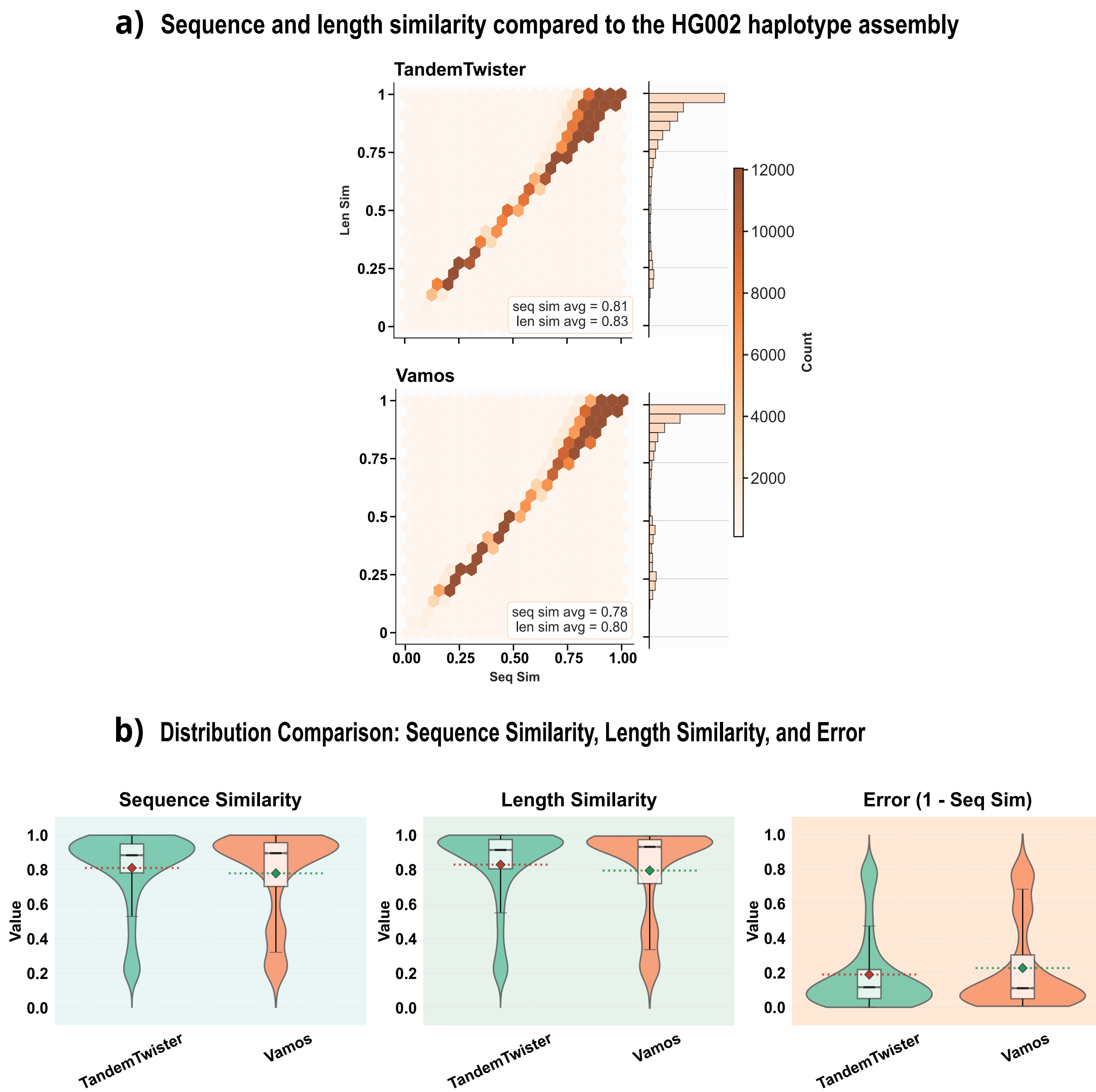

Supplementary Figure S4: Sequence accuracy on Oxford Nanopore (ONT) sample HG002. a) Comparison of the concatenated TR allele sequences with the high-quality haplotype assemblies. Hexa plots representing density of existing regions with various sequence and length similarities in the two tools. b) Comparison of different tool's distributions of sequence similarity (left), length similarity (middle) and sequence error rate (right).

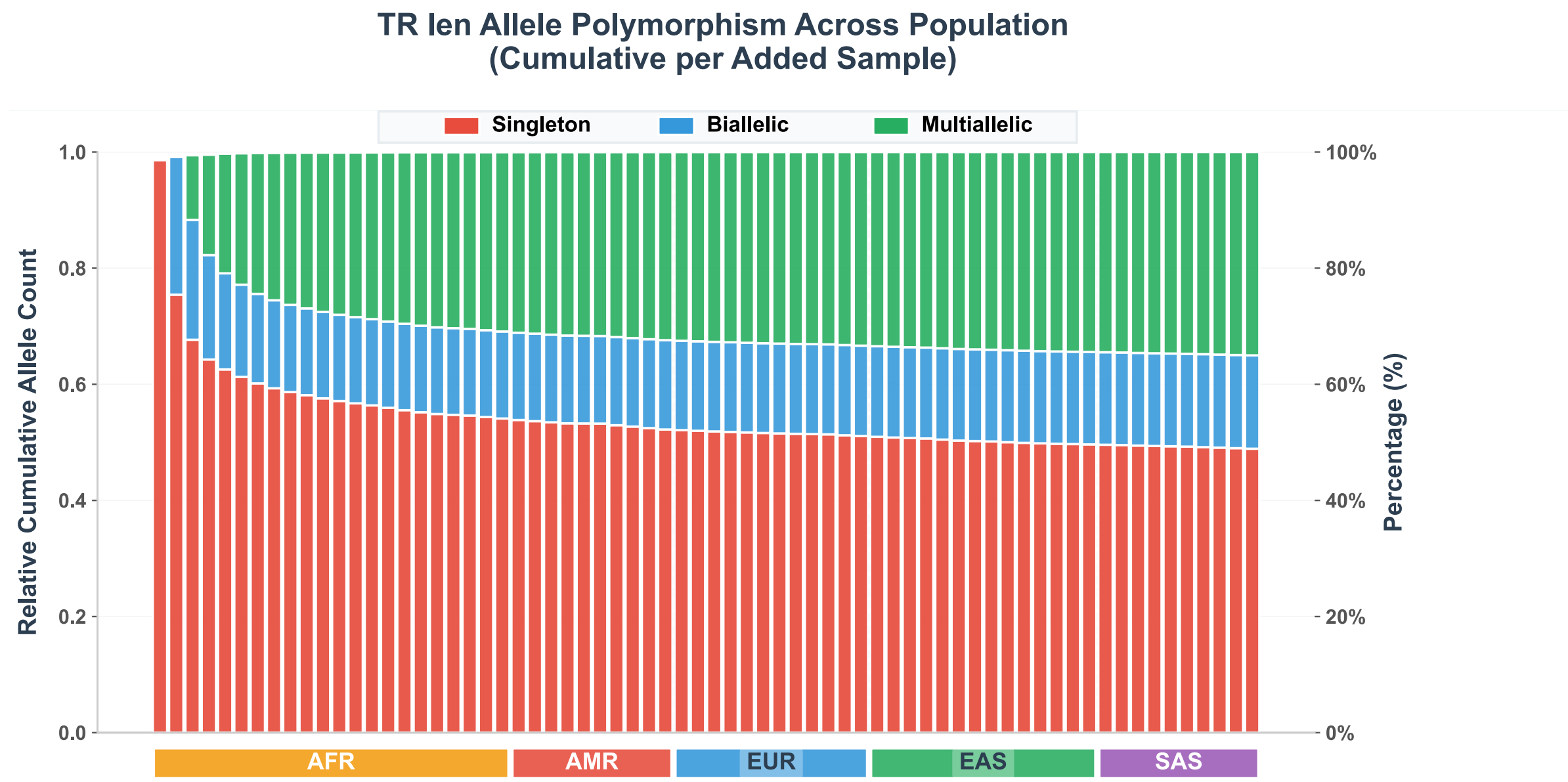

Supplementary Figure S5: Accumulative plots on tandem repeat variation by length in population. The TR regions are divided into three categories by their length variation: Singleton (red), biallelic (blue), and multi-allelic (green). The bar plot shows the cumulative portion of these categories of tandem repeats in population by adding samples one by one in the displayed order of super-populations.

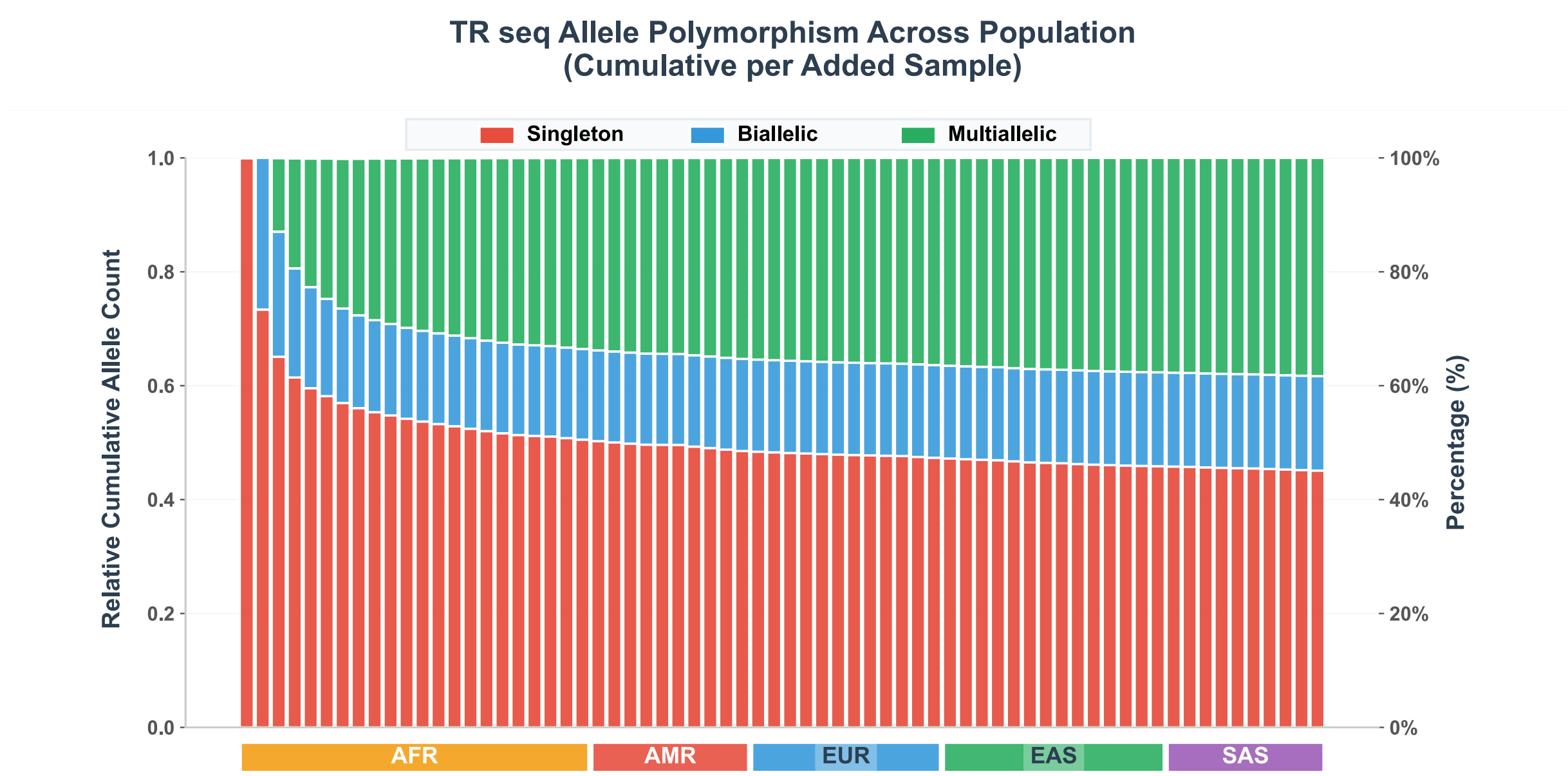

Supplementary Figure S6: Accumulative plots on tandem repeat variation by sequence in population. The TR regions are divided into three categories by their sequence variation: Singleton (red), biallelic (blue), and multi-allelic (green). The bar plot shows the cumulative portion of these categories of tandem repeats in population by adding samples one by one in the displayed order of super-populations.

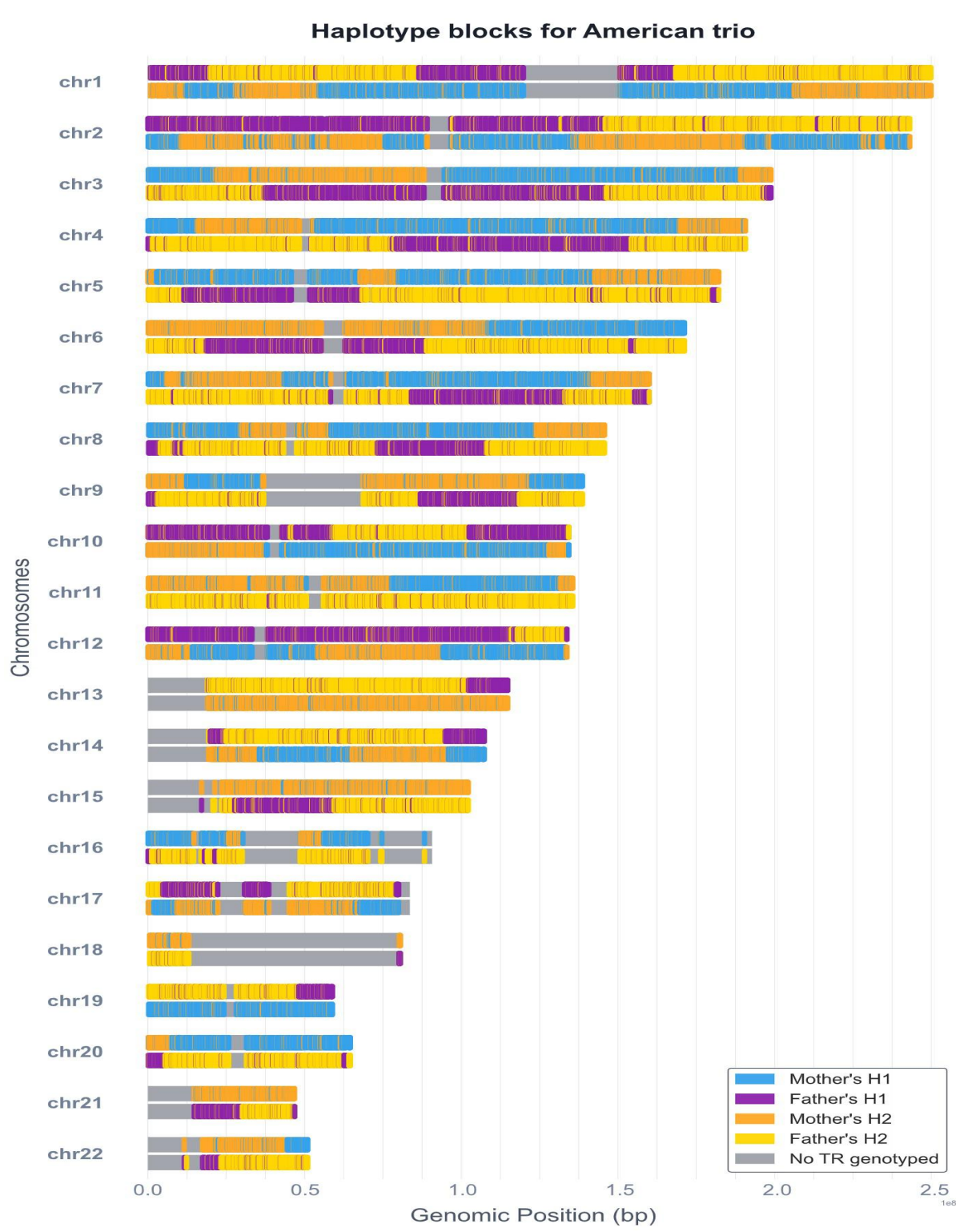

(a) Mixed American trio inheritance pattern

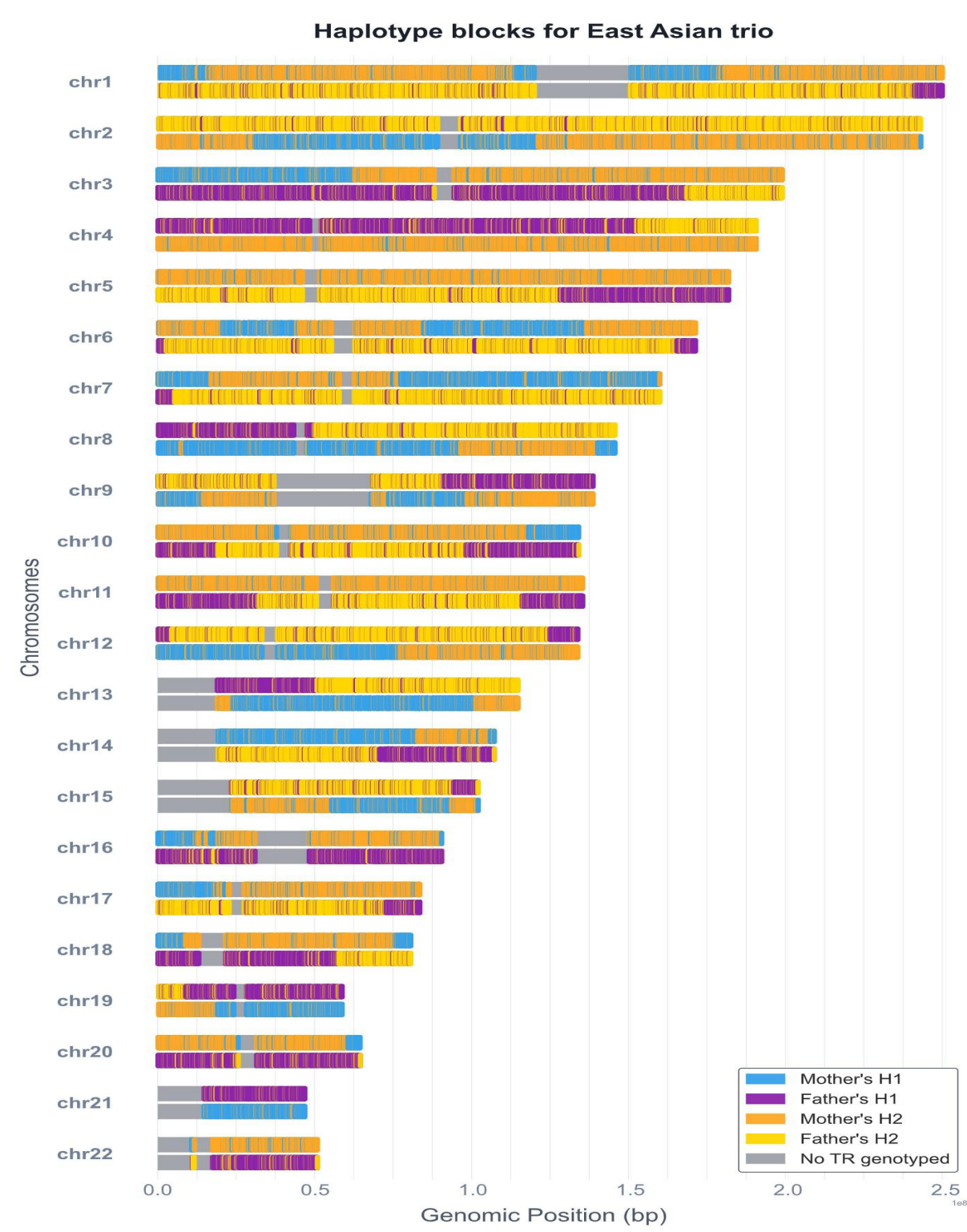

(b) East Asian trio inheritance pattern

Supplementary Figure S7: Inherited haplotype alleles from parents in different population trios. a) Admixed American (AMR) trio, b) Ease Asian (EAS) trio. Different colors refer to the mother or father alleles that are inherited to the child haplotype h1 or h2 (four possible combinations)

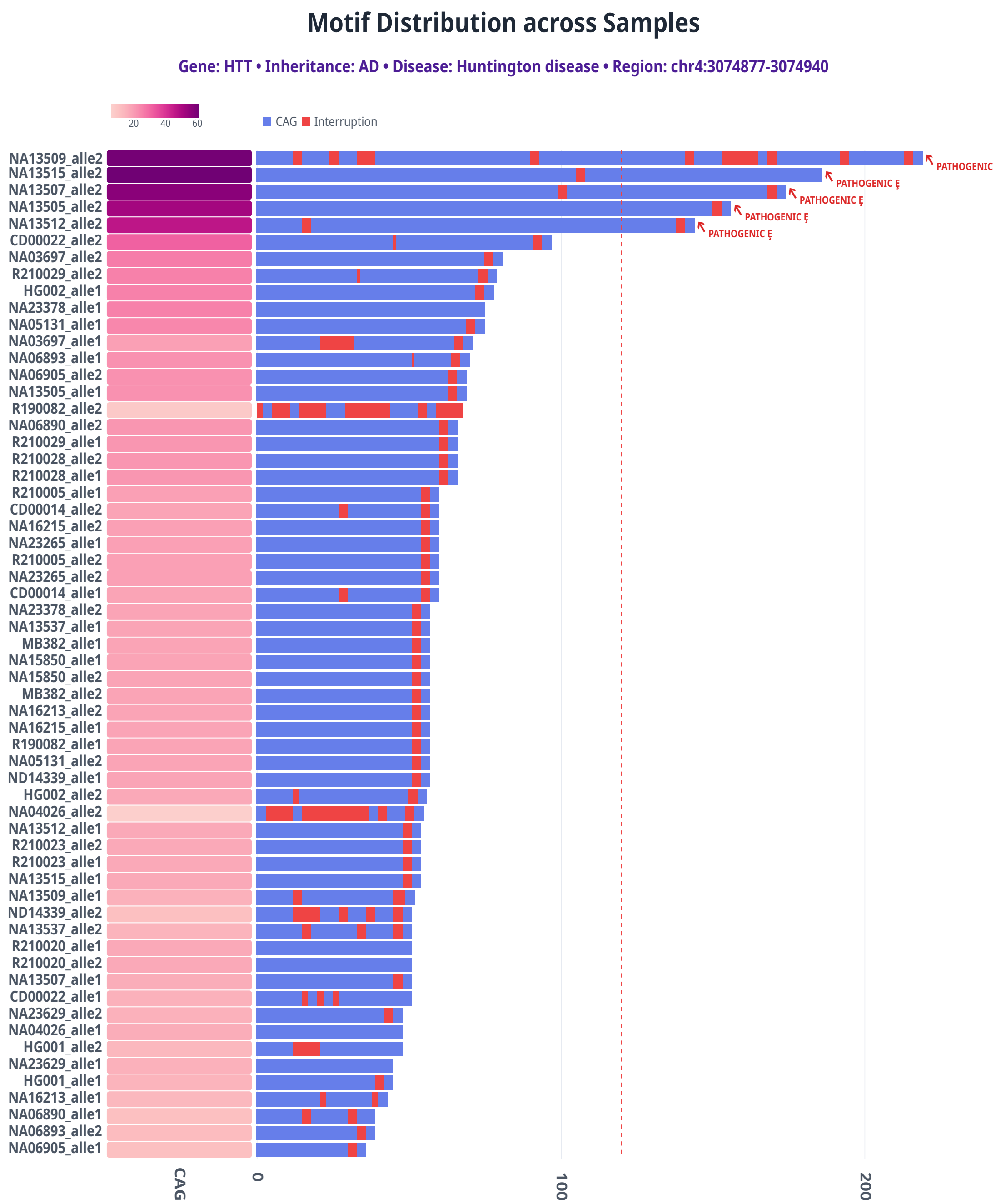

Supplementary Figure S8: TandemTwister genotyping results for HTT gene across 31 samples. Left: Heatmap of motif counts; Right: Bar plot showing copy number alleles per sample. Red dashed line indicates pathogenic cutoff. Five samples are affected by the disease and show pathogenic expansion.

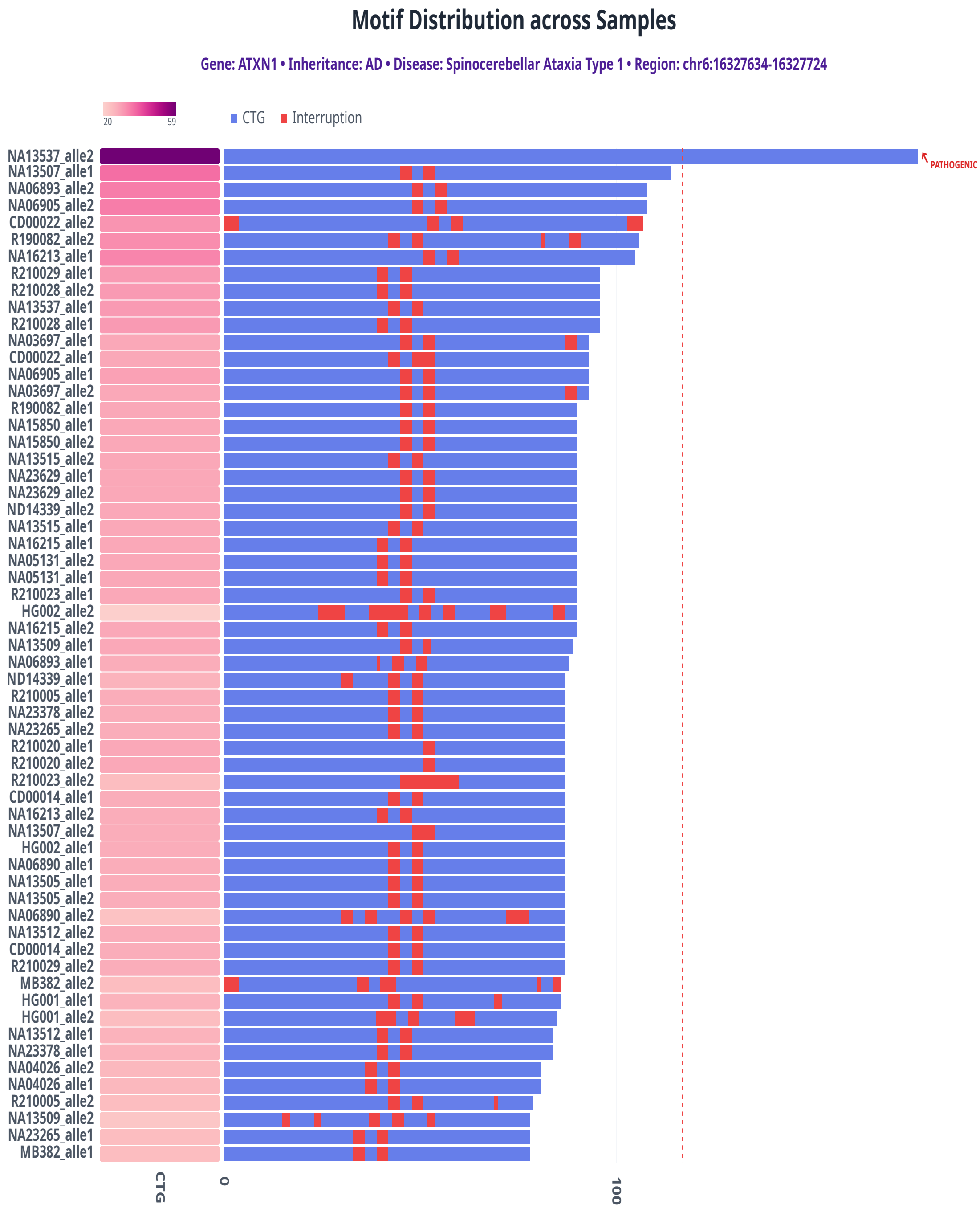

Supplementary Figure S9: TandemTwister genotyping results for ATXN1 gene across 31 samples. Left: Heatmap of motif counts; Right: Bar plot showing copy number alleles per sample. Red dashed line indicates pathogenic cutoff. Sample NA13537 shows pathogenic expansion.

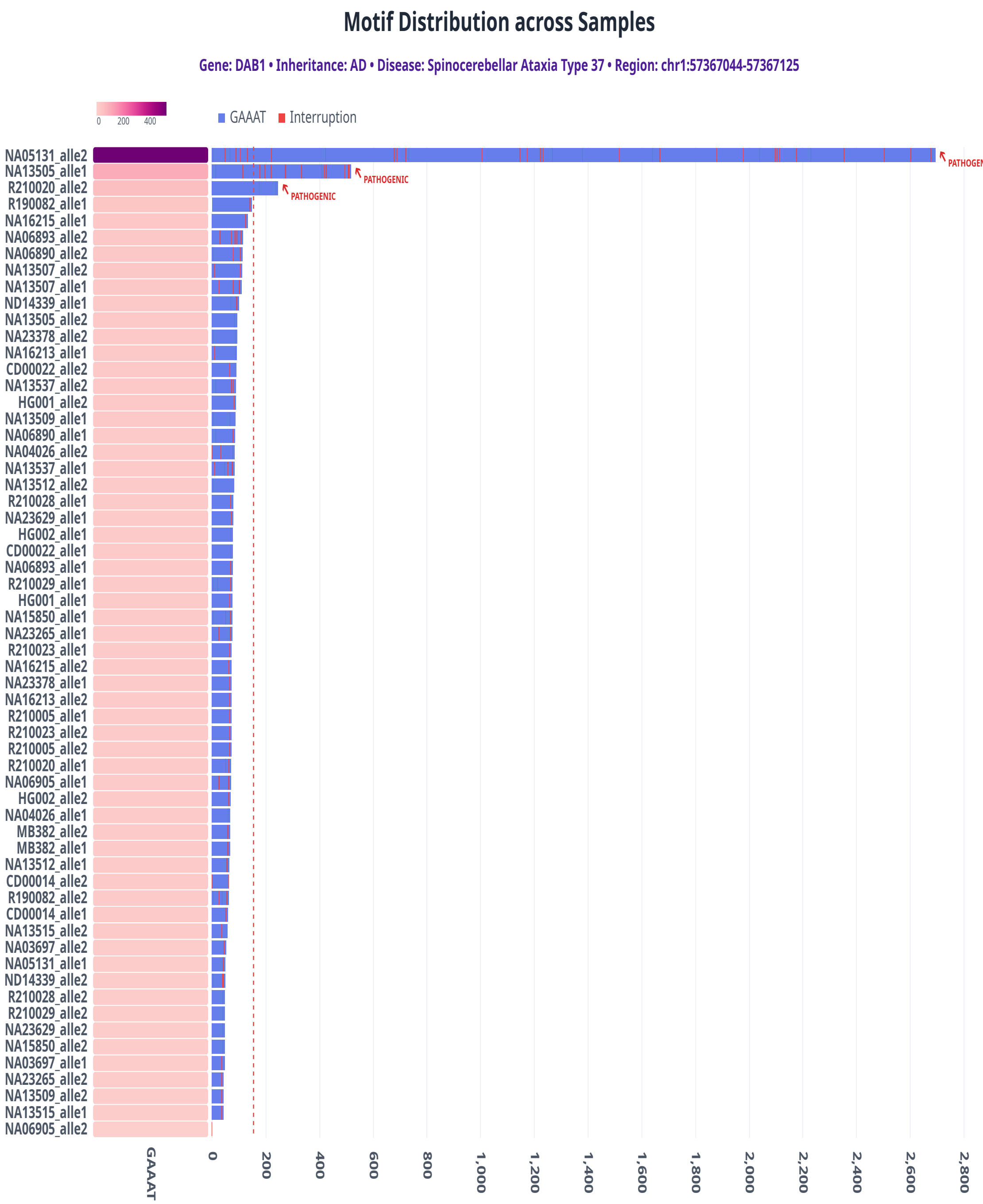

Supplementary Figure S10: TandemTwister genotyping results for DAB1 gene across 31 samples. Left: Heatmap of motif counts; Right: Bar plot showing copy number alleles per sample. Red dashed line indicates pathogenic cutoff. Three samples are affected by the disease and show pathogenic expansion.

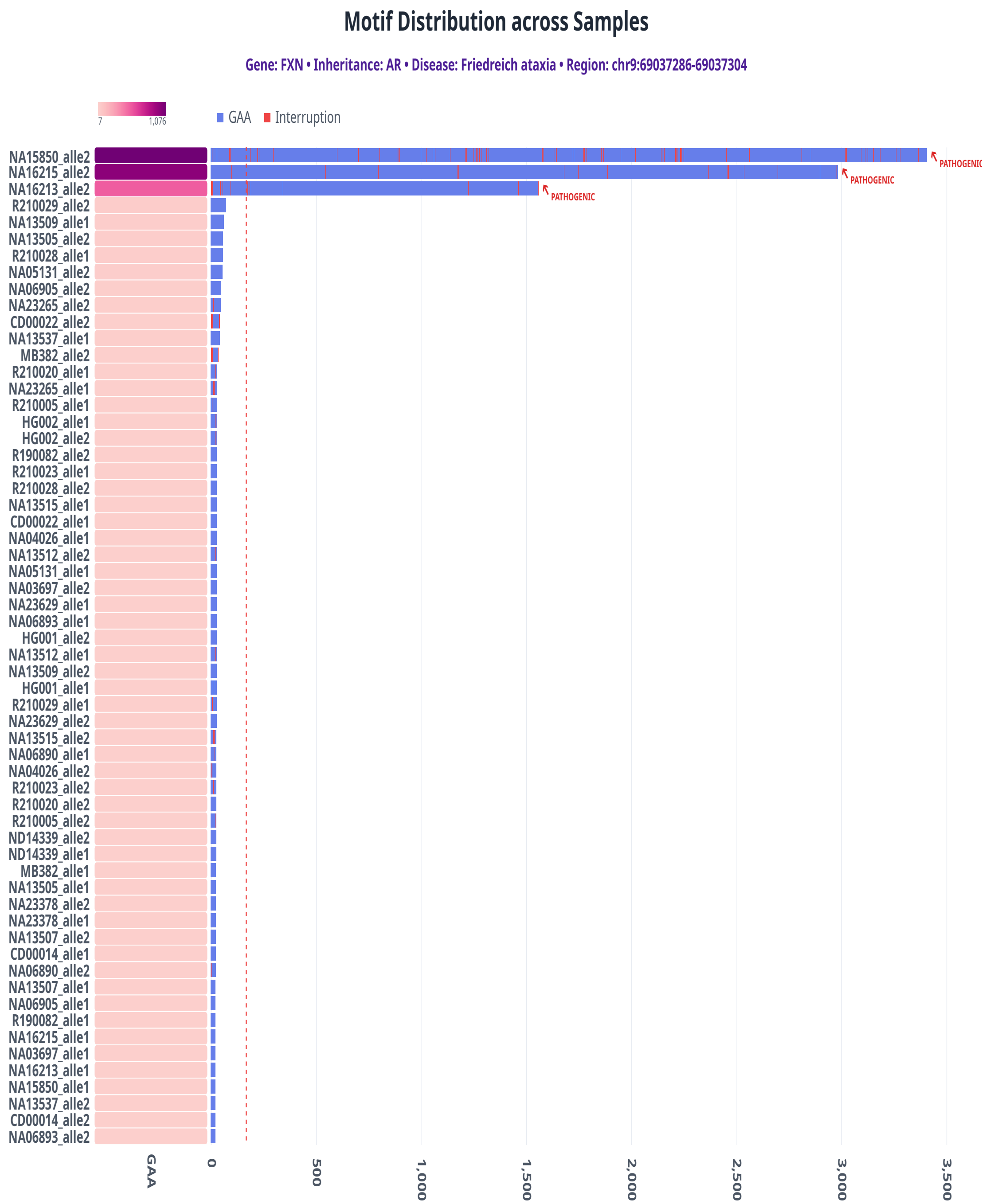

Supplementary Figure S11: TandemTwister genotyping results for FXN gene across 31 samples. Left: Heatmap of motif counts; Right: Bar plot showing copy number alleles per sample. Red dashed line indicates pathogenic cutoff. Three samples exhibit pathogenic expansions above the cutoff.

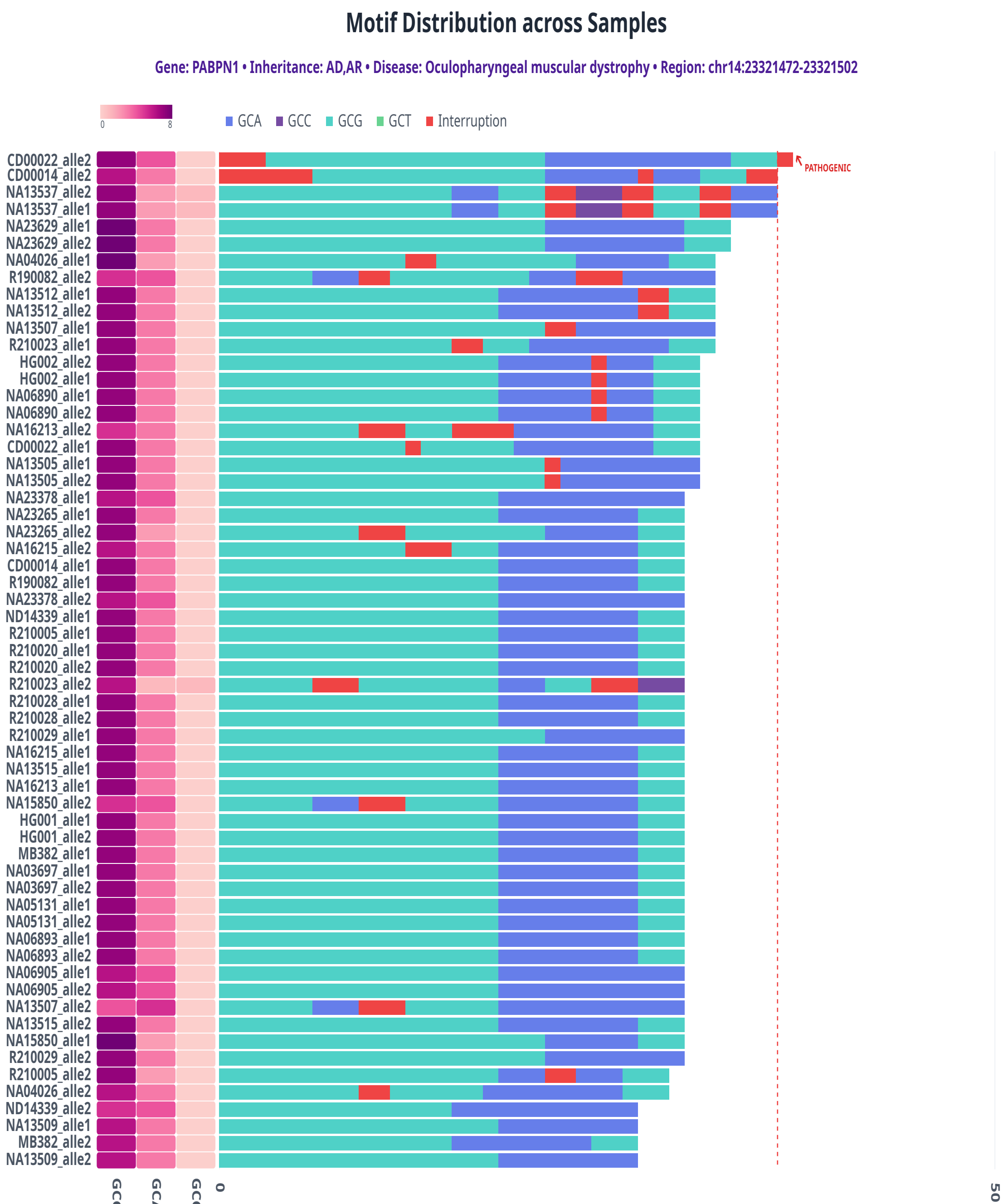

Supplementary Figure S12: TandemTwister genotyping results for PABPN1 gene across 31 samples. Left: Heatmap of motif counts; Right: Bar plot showing copy number alleles per sample. Red dashed line indicates pathogenic cutoff. Sample CD00022 shows pathogenic expansion.

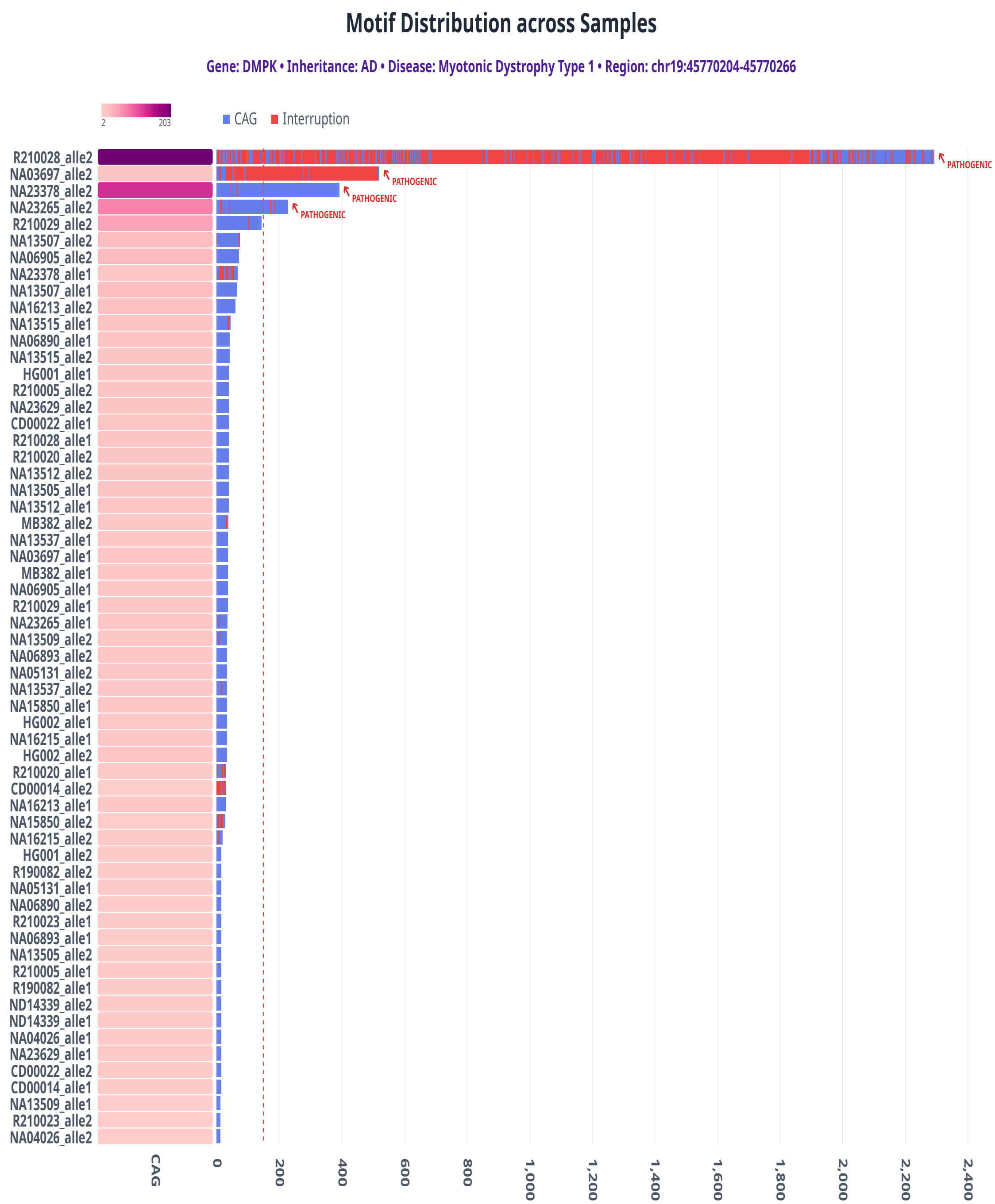

Supplementary Figure S13: TandemTwister genotyping results for DMPK gene across 31 samples. Left: Heatmap of motif counts; Right: Bar plot showing copy number alleles per sample. Red dashed line indicates pathogenic cutoff. Four samples are affected by the disease and show pathogenic expansion.

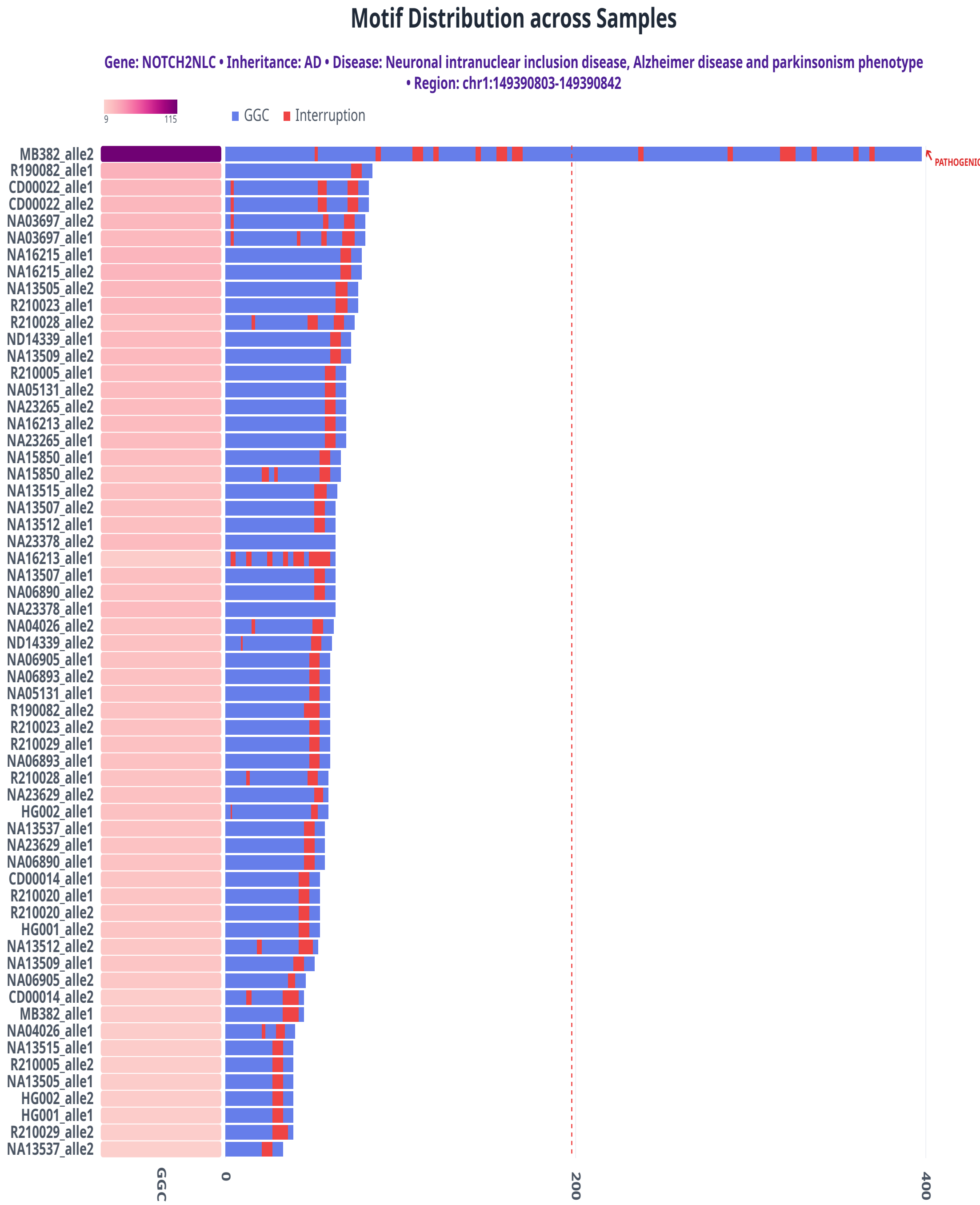

Supplementary Figure S14: TandemTwister genotyping results for NOTCH2NLC gene across 31 samples. Left: Heatmap of motif counts; Right: Bar plot showing copy number alleles per sample. Red dashed line indicates pathogenic cutoff. Sample MB382 shows pathogenic expansion.

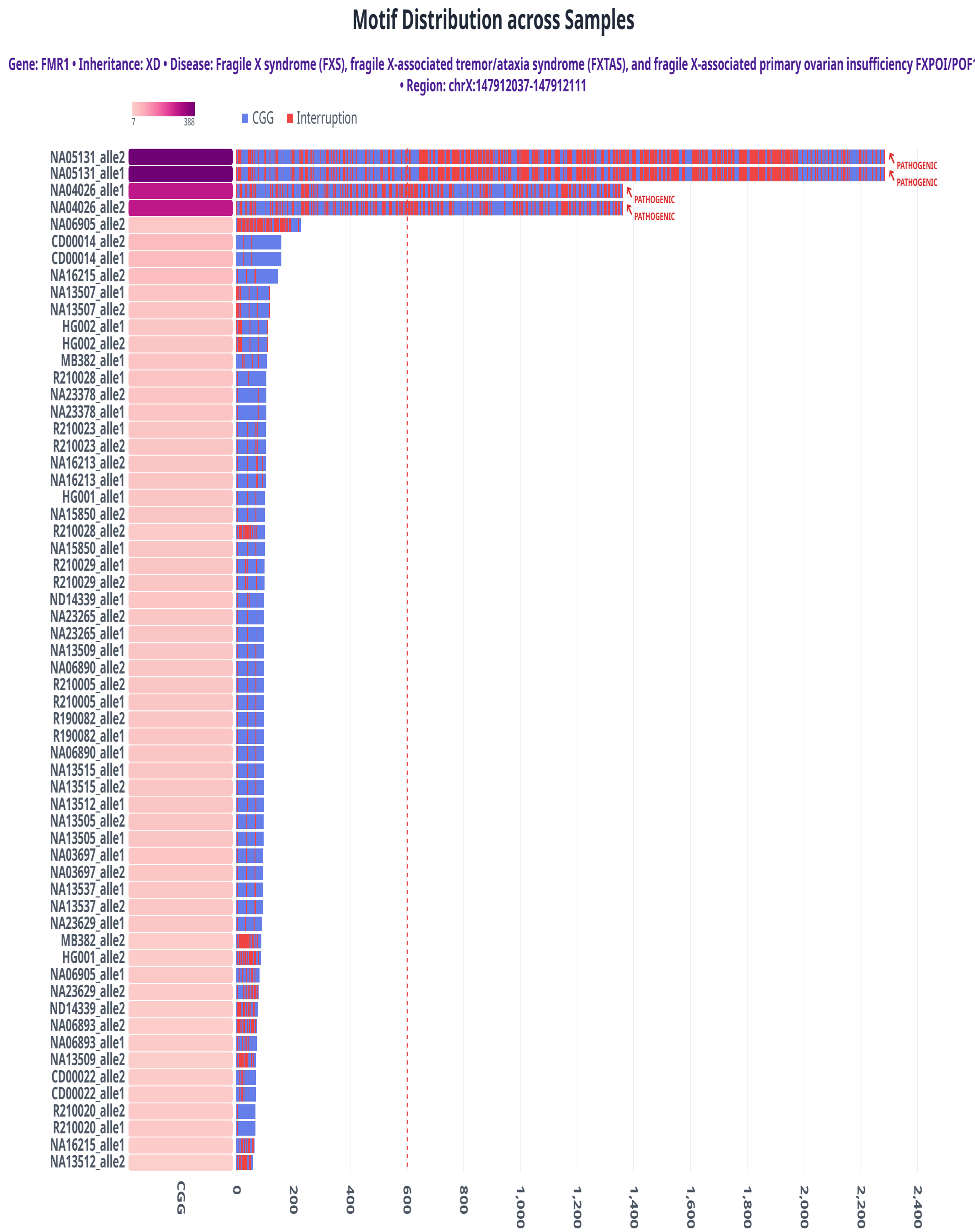

Supplementary Figure S15: TandemTwister genotyping results for FMR1 gene. Two samples show pathogenic allele expansions. TandemTwister genotyping results for FMR1 gene across 31 samples. Left: Heatmap of motif counts; Right: Bar plot showing copy number alleles per sample. Red dashed line indicates pathogenic cutoff. Two samples (four alleles) show pathogenic allele expansions.

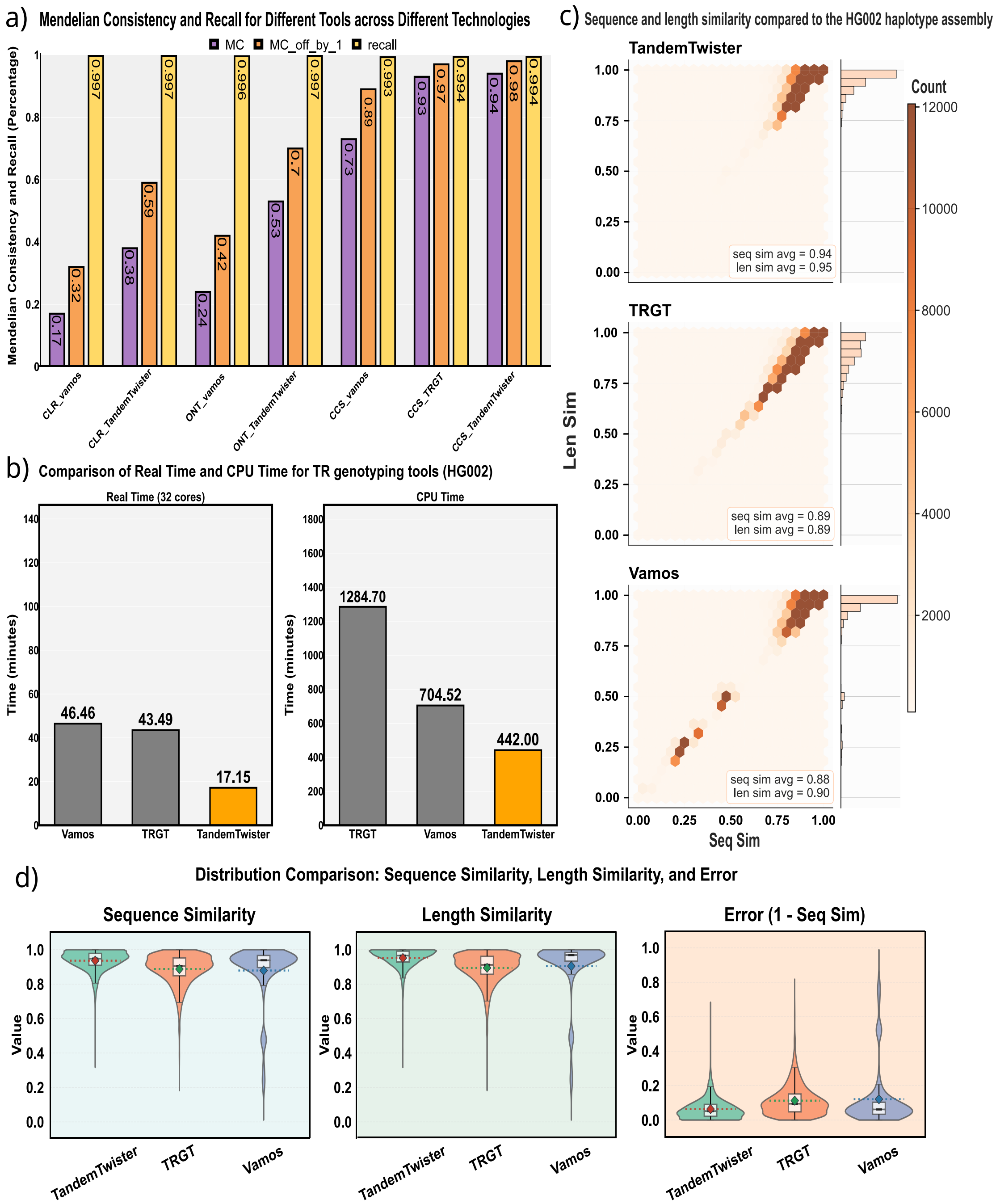

Supplementary Figure S16: Evaluation of TR genotyping tools. a) Mendelian consistency (MC) and recall values for different long-read sequencing technologies and the older versions TR genotyping tools (Vamos 2.1.7, TRGT 4.0.0). These tools versions were the most recent versions in time of our initial manuscript submission. Purple and Orange bars show the default and off-by-one Mendelian consistencies in the Ashkenazi trio, respectively, and yellow bars represent the recall values (fraction of the genotyped regions) for the child sample HG002. b) bar plot illustration of running time performance comparison of the tools based on their wall-clock time (using 32 cores) and CPU running time. The fastest genotyping tool is *TandemTwister*. c) Comparison of the concatenated TR allele sequences with the high-quality haplotype assemblies. The analysis is for the CCS sample HG002 and different subplots show performances of various tools. Each subplot shows the length and sequence similarity distribution across all TR regions per tool and sequencing technology. d) Comparison of different tool's distributions of sequence similarity (left), length similarity (middle) and sequence error rate (right).
